# Systematic Analysis of Immune Infiltration and Predicting Prognosis in Clear Cell Renal Cell Carcinoma Based on the Inflammation Signature

**DOI:** 10.3390/genes13101897

**Published:** 2022-10-19

**Authors:** Yuke Zhang, Chunliu Shi, Yue Chen, Hongwei Wang, Feng Chen, Ping Han

**Affiliations:** 1 Department of Urology, West China Hospital, Sichuan University, Chengdu 610041, China; 2 Department of Orthopedics, The Second People’s Hospital of Deyang, Deyang 618000, China; 3 Department of Gastrointestinal and Vascular Surgery, Guanghan People’s Hospital, Guanghan 618300, China; 4 Department of Obstetrics and Gynecology, Chongzhou Maternal and Child Health Care Hospital, Chongzhou 611230, China; 5 Department of Integrated Care Management Center, West China Hospital, Sichuan University, Chengdu 610041, China; 6 West China School of Nursing, Sichuan University, Chengdu 610041, China

**Keywords:** clear cell renal cell carcinoma, inflammation, risk signature, tumor microenvironment, drug molecules

## Abstract

Clear cell renal cell carcinoma (ccRCC) is the most frequent kind of kidney malignancy. Inflammation is a physiological response of the immune system to harmful stimuli. Notably, the role inflammation plays in ccRCC is still unknown. In this study, consensus clustering analysis sorted the ccRCC specimens from the TCGA dataset into C1 and C2 clusters. The C2 cluster comprised ccRCC specimens with a high TNM stage and tumor grade. These specimens were characterized by the activation of the inflammatory response and an immunosuppressive microenvironment. A seven-gene inflammation-related risk signature was designed employing the LASSO and Cox regression analyses for the inflammation-related genes. The ccRCC specimens were classified into two groups with high and low risk by calculating the risk scores. The specimens in the group with high risk showed a poor prognosis and were positively correlated with immune inhibitory factors. Moreover, a nomogram was created by incorporating inflammation-related risk signatures and clinical characteristics. The ROC and DCA curves indicated a satisfactory efficiency of the nomogram for predicting the survival outcomes. Furthermore, we identified the potential therapeutic drug molecules through CMap analysis. The findings of our study may act as a guide for further research on new prognostic biomarkers and therapies.

## 1. Introduction

Renal cell carcinoma (RCC) is a lethal cancer with a high prevalence, accounting for nearly 2% of new cancer cases and deaths worldwide [1]. It comprises three primary histological subtypes—clear cell RCC (ccRCC), papillary RCC, and chromophobe RCC—of which ccRCC is the most frequent and contributes to approximately 75% of RCC incidence [2]. Early-stage localized ccRCC can be treated with surgery; however, in approximately 30% of the cases, the disease eventually progresses to metastasis [3]. Recently, targeted therapy and immunotherapy have proved successful in the clinical treatment for advanced-stage ccRCC and were shown to prolong the survival of patients to an extent; however, the therapeutic responses are variable, and patients’ prognoses are generally poor [4]. Therefore, the investigation of new prognostic biomarkers and therapeutic strategies is necessary.

Inflammation is a defensive response of the immune system to harmful stimuli, such as tissue disruption, infection, and toxins [5]. It induces the activation and recruitment of innate and adaptive immune cells to maintain tissue homeostasis [6]. The renowned pathologist Rudolf Virchow first proposed a potential relationship between inflammation and cancer, indicating that the site of chronic inflammation can show cancer development [7]. Evidence from numerous studies has validated Virchow’s hypothesis, and inflammation has been regarded as a hallmark of cancer [8]. When tissues undergo injury or infection, inflammation is initiated for tissue repair, and inflammation resolves after the repair is completed or the infection subsides [9]. However, inflammation associated with cancer development does not resolve [10]. Persistent inflammation accumulates various chemokines, cytokines, growth factors, and signaling molecules in the tumor microenvironment (TME). The inflammatory TME induces the proliferation and metastasis of cancer cells, which is essential for therapy resistance via activating oncogenes and restraining tumor suppressor genes [11]. Notably, the role inflammation plays in ccRCC is still unknown. Hence, a comprehensive understanding of inflammation can help investigate the pathogenesis of ccRCC and develop novel therapeutic methods.

In this study, we classified 528 ccRCC specimens into two inflammation clusters using consensus clustering analysis. Two clusters showed different survival outcomes, clinical characteristics, and immune microenvironments. An inflammation-related risk signature (IRRS) was constructed following the expression of seven inflammation-related genes. The IRRS exhibited satisfactory predictive performance for patients’ prognoses. We further developed a nomogram by incorporating the IRRS and clinical parameters to improve predictive accuracy.

## 2. Materials and Methods

### 2.1. Data Collection

Gene expression data of raw count and transcripts per million (TPM) of 72 healthy kidney tissues and 541 ccRCC specimens were taken from The Cancer Genome Atlas (TCGA) database (https://portal.gdc.cancer.gov/, accessed on 13 April 2022). After removing ccRCC specimens having a follow-up duration of less than one day, 528 ccRCC specimens were incorporated into this study. The simple nucleotide variation (SNV) data were also retrieved from the TCGA database. The E-MTAB-1980 dataset was retrieved from the ArrayExpress database (https://www.ebi.ac.uk/arrayexpress/, accessed on 27 December 2021), which contained the microarray and clinical data of 101 ccRCC specimens. The Checkmate dataset included information from 130 patients with ccRCC receiving everolimus and 181 patients receiving nivolumab.

### 2.2. Gene and Pathway Functional Enrichment Analysis

Gene ontology (GO) and Kyoto Encyclopedia of Genes and Genomes (KEGG) pathway terms were used for determining the gene function. GO is used for characterizing the biological roles of genes in three broad categories: biological process (BP), cellular component (CC), and molecular function (MF). Thirty-seven BP terms were related to inflammation. The “c5.go.bp.v7.5.1.symbols” was taken from Molecular Signatures Database (MSigDB, http://www.gsea-msigdb.org/gsea/msigdb, accessed on 23 June 2022). The ssGSEA algorithm, in accordance with the “GSVA” R package, was utilized to calculate the enrichment scores of inflammation-related terms [12]. The “clusterProfiler” R package was employed to carry out GO and KEGG pathway analyses for genes associated with inflammation [13].

### 2.3. Identification of Inflammation-Related Genes

Genes associated with inflammation were retrieved from 37 inflammation-related BP terms. A total of 765 protein-coding inflammation-related genes were identified. Subsequently, the “edgeR” software was used to examine the expression profiles of raw count [14]. The threshold of log2|fold change (FC)| > 2 and false discovery rate (FDR) < 0.05 were taken as the screening criteria for inflammation-related differentially expressed genes (DEGs) between healthy kidney specimens and ccRCC specimens. Additionally, the DEGs between C1 and C2 clusters were screened out using these genes. FDR < 0.05 was set as the threshold.

### 2.4. Estimation of the Tumor Mutation Burden (TMB) in ccRCC

The SNV data were analyzed using “maftools” to identify the SNVs of genes in each ccRCC specimen [15]. In addition, the TMB of the ccRCC specimens was calculated by using the “tmb” algorithm.

### 2.5. Consensus Clustering Analysis for Senescence-Related Genes

Based on the enrichment scores of 37 inflammation-related BP terms, the “ConsensusClusterPlus” R package was utilized to classify 528 ccRCC specimens into two inflammation clusters employing the “Pearson” algorithm and 1000 bootstraps [16]. The consensus cumulative distribution function (CDF) curves were utilized to choose the optimum number of clusters.

### 2.6. Estimation of the Tumor Immune Signatures and Inhibitory Immune Checkpoint Molecules

The “IOBR” R package was utilized to evaluate the tumor immune signatures, such as immune exhaustion, immune suppression, and immune microenvironment [17]. In addition, the variations in the expression levels of ten representative inhibitory molecules of immune checkpoint between the two risk groups were determined.

### 2.7. Construction and Validation of the IRRS

The TCGA dataset was utilized for constructing the IRRS, and the E-MTAB-1980 dataset was employed as an external validation dataset. The Checkmate dataset was used for verifying the predictive performance of the IRRS for the prognosis of ccRCC in patients receiving targeted treatment or immunotherapy. To this end, the batch effect among the TCGA, E-MTAB-1980, and Checkmate datasets was removed with the help of the “ComBat” algorithm. The inflammation-related genes were used in LASSO and multivariate Cox regression analyses to identify the final genes and the corresponding regression coefficients involved in constructing the IRRS. The risk score was calculated through the following formula: coefficient gene (a) × gene expression (a) + coefficient gene (b) × gene expression (b) + … + coefficient gene (n) × gene expression (n). The ccRCC specimens were classified into high- and low- risk groups by following the median IRRS-based risk scores. The variations in the overall survival (OS) or progression-free survival (PFS) between both the risk groups were assessed using the Kaplan–Meier (K–M) survival curves with the “survival” R package [18]. The receiver operating characteristic (ROC) curves produced by the “timeROC” R package were employed for estimating the areas under the curves (AUCs) [19]. Principal component analysis (PCA), uniform manifold approximation and projection (UMAP), and t-distributed stochastic neighbor embedding (t-SNE) were carried out using “stats”, “umap”, and “Rtsne” R packages, respectively. The external E-MTAB-1980 dataset was utilized for carrying out the analyses as mentioned earlier to validate the reliability of the IRRS.

### 2.8. Development and Validation of a Nomogram

The TCGA dataset was utilized for constructing a nomogram by incorporating the IRRS and the clinical parameters using the “rms” and “regplot” R packages. The calibration curves helped find the uniformity in the anticipated and actual survival outcomes. The anticipated accuracies of the nomogram, IRRS, and clinical characteristics were assessed using ROC and decision curve analysis (DCA) curves. The analyses as mentioned earlier were conducted on the E-MTAB-1980 dataset to confirm the nomogram efficacy.

### 2.9. Identification of Potential Drug Molecules

The DEGs between IRRS-classified high- and low-risk groups were detected based on the threshold of log2|FC| > 1.5 and FDR < 0.05. Using the CMap database (https://clue.io/, accessed on 15 August 2022), the common up-regulated DEGs between the TCGA and E-MTAB-1980 datasets were analyzed to identify potential drug molecules.

### 2.10. Statistical Analysis

Analyses were conducted through R (version 4.1.3). The Chi-square test was employed to assess the variations in the composition of clinical parameters between the inflammation-related clusters or risk groups. The variation in the tumor immune signatures, immune cell infiltration, and TMB between both groups were assessed utilizing the Wilcoxon test. The relationship between the two variables was assessed using Pearson’s correlation coefficient. A *p*-value < 0.05 was taken as a statistically significant value.

## 3. Results

### 3.1. Estimation of Inflammation-Related Signatures between Healthy Kidney Tissues and ccRCC Specimens

**Figure 1:**
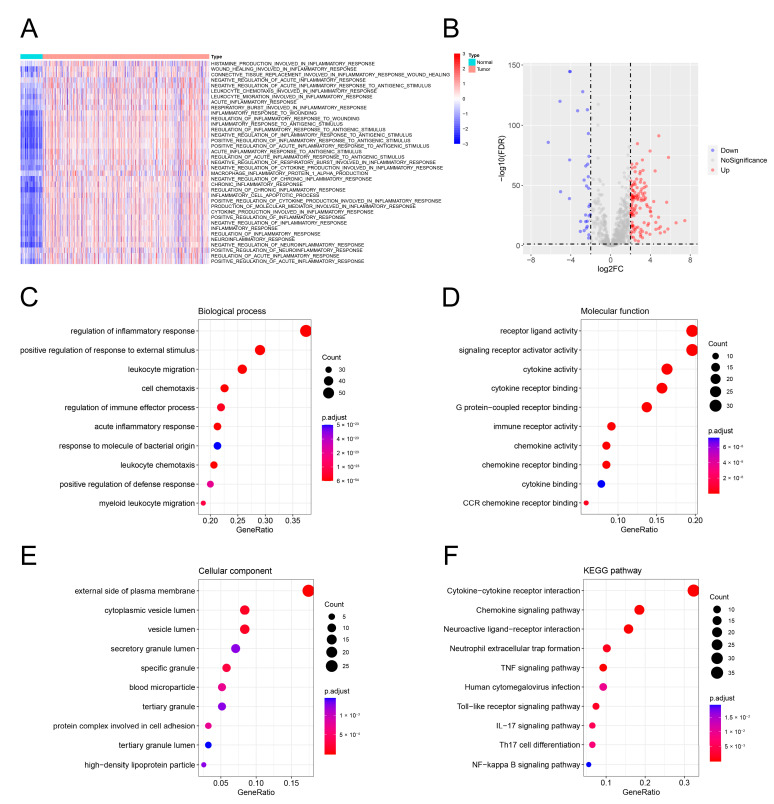
Inflammation-related biological functions were activated in ccRCC. (**A**) Functional enrichment of 37 inflammation-related BP terms in healthy kidney tissues and ccRCC tissues. (**B**) Inflammation-related DEGs between healthy and ccRCC kidney tissues. (**C**–**F**) Functional enrichment of inflammation-related DEGs by GO and KEGG pathway analyses.

The enrichment scores of 37 inflammation-related BP terms were calculated using the ssGSEA algorithm. The results indicated that the inflammation-related signatures were primarily up-regulated in ccRCC (Figure 1A). Subsequently, 155 inflammation-related DEGs were identified based on the threshold of log2|FC| > 2 and FDR < 0.05 (Figure 1B). The biological roles of the 155 DEGs associated with inflammation were investigated by the analysis of GO and KEGG terms. The terms from BP, MF, and CC were primarily enriched in “regulation of inflammatory response”, “receptor ligand activity”, and “external side of plasma membrane”, respectively (Figure 1C–E). In the KEGG pathway, the genes were related to “cytokine-cytokine receptor interaction” (Figure 1F).

### 3.2. Identification of Two Inflammation Clusters

**Figure 2:**
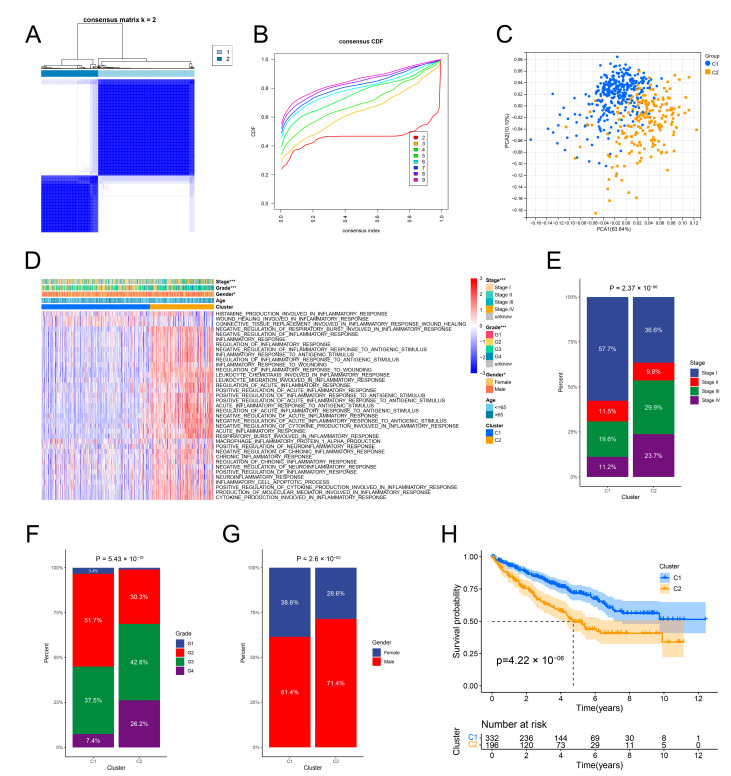
Identification of inflammation clusters of ccRCC based on inflammation-related signatures. (**A**) Consensus cluster matrix for k = 2. (**B**) CDF curves for k = 2–9. (**C**) PCA for two inflammation clusters. (**D**) Functional enrichment of 37 inflammation-related BP terms and distribution of clinical parameters between two inflammation clusters. (**E**–**G**) The two inflammation clusters differ in the proportions of the tumor stage, tumor grade, and gender components. (**H**) OS of the two inflammation clusters. * *p* < 0.05; and *** *p* < 0.001.

The ccRCC specimens in the TCGA dataset were classified into C1 (*n* = 332) and C2 (*n* = 196) clusters by consensus clustering for 37 inflammation-related signatures (Figure 2A). The CDF curves confirmed that the best number of clusters was two (Figure 2B). The PCA plot demonstrated that the ccRCC specimens from various clusters were distributed in distinct sections (Figure 2C). The inflammation-related signatures were highly activated in the C2 cluster (Figure 2D). It was observed that the proportion of advanced TNM stage and tumor-grade ccRCC specimens and male patients was higher in the C2 cluster in contrast with the C1 cluster (Figure 2E–G). According to the K–M survival curves, the prognosis of ccRCC in the C2 cluster was weaker compared to the C1 cluster (Figure 2H).

### 3.3. Different TME Characteristics between the Two Clusters

**Figure 3:**
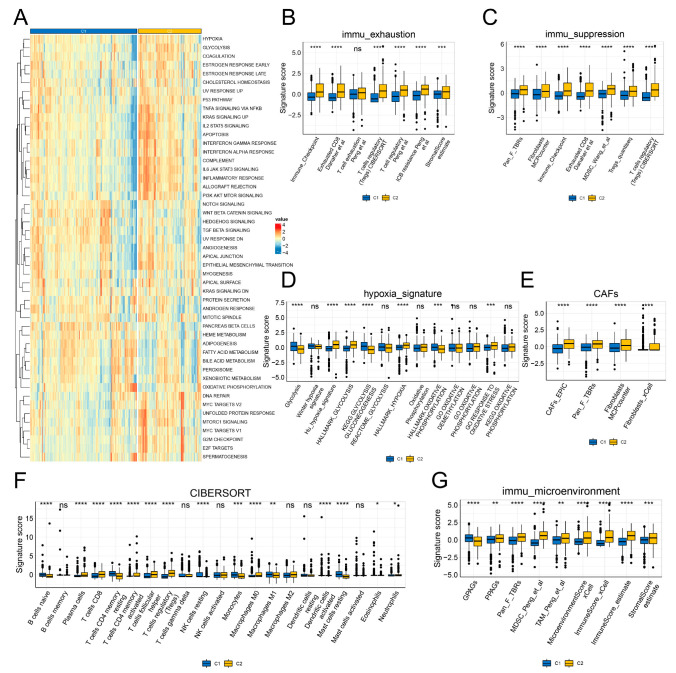
Immune characteristics of the two inflammation clusters. (**A**) Cancer hallmarks of the two inflammation clusters. (**B**) Immune exhaustion signature of the two inflammation clusters. (**C**) Immune suppression signature of the two inflammation clusters. (**D**) Hypoxia signature of the two inflammation clusters. (**E**) CAFs signature of the two inflammation clusters. (**F**) Infiltration levels of immune cells between the two inflammation clusters were estimated using the CIBERSORT algorithm. (**G**) Immune microenvironment signature of the two inflammation clusters. * *p* < 0.05; ** *p* < 0.01; *** *p* < 0.001; and **** *p* < 0.0001.

We evaluated the hallmarks of cancer in the two inflammation clusters. Inflammation-related hallmarks, such as TNF-α, KRAS, IL2/STAT5, and IL6/JAK/STAT3 signaling, inflammatory response, and complement, were remarkably up-regulated in the C2 cluster. In contrast, several metabolism-related hallmarks, including adipogenesis, peroxisomes, and heme and fatty acid metabolisms were down-regulated in the C2 cluster (Figure 3A). The microenvironment of the C2 cluster showed an immune-exhausted and -suppressed phenotype (Figure 3B,C). As hypoxia and cancer-associated fibroblasts (CAFs) are associated with inflammation and play important roles in oncogenesis, we estimated differences in the hypoxia- and CAF-related signatures between the two clusters. The hypoxia- and CAF-related signatures were enhanced in the C2 cluster (Figure 3D,E). Moreover, higher infiltration levels of plasma cells, CD8 T cells, activated CD4 memory T cells, follicular helper T cells, regulatory T cells (Tregs), M0 macrophages, and neutrophils were observed in the C2 cluster as compared to the C1 cluster. The C1 cluster showed higher infiltration levels of naive B cells, resting CD4 memory T cells, resting NK cells, monocytes, M1 macrophages, activated dendritic cells, resting mast cells, and eosinophils (Figure 3F). In addition, the C2 cluster showed an up-regulation of the immune microenvironment signature scores related to poor-prognosis angiogenesis genes (PPAGs), pan-fibroblast TGF-β response signature (Pan-F-TBRS), and myeloid-derived suppressor cells (MDSCs), as well as the immune, stromal, and microenvironment scores. Good-prognosis angiogenesis genes (GPAGs) were down-regulated in the C2 cluster (Figure 3G).

### 3.4. Construction of the IRRS

**Figure 4:**
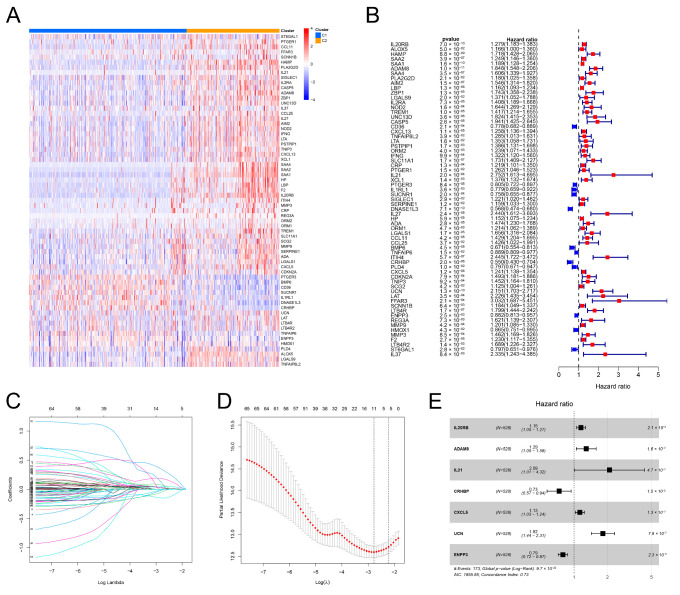
Development of an IRRS. (**A**) Heatmap of inflammation-related DEGs between the two inflammation clusters. (**B**) Outcomes of the univariate Cox regression analysis of inflammation-related DEGs. (**C**,**D**) Lambda. min value = 11 was determined through LASSO regression analysis. (**E**) Outcomes of multivariate Cox regression analysis.

As mentioned above, differential expression of 155 genes associated with inflammation was observed between healthy kidney tissues and ccRCC specimens. Differential expression and univariate Cox regression analyses were carried out using these genes to identify inflammation-related prognostic DEGs between the two clusters. We identified 65 genes (Figure 4A,B). LASSO regression analysis was conducted for 65 genes to yield 11 genes (Figure 4C,D). These genes were further analyzed using the multivariate Cox regression analysis. Seven genes and the corresponding regression coefficients were identified to construct the IRRS (Figure 4E). The risk scores were computed based on the formula: 0.140 × L20RB + 0.247 × ADAM8 + 0.715 × IL21 − 0.302 × CRHBP + 0.116 × CXCL5 + 0.583 × UCN − 0.227 × ENPP3.

### 3.5. Validation of the Predictive Performance of the IRRS

**Figure 5:**
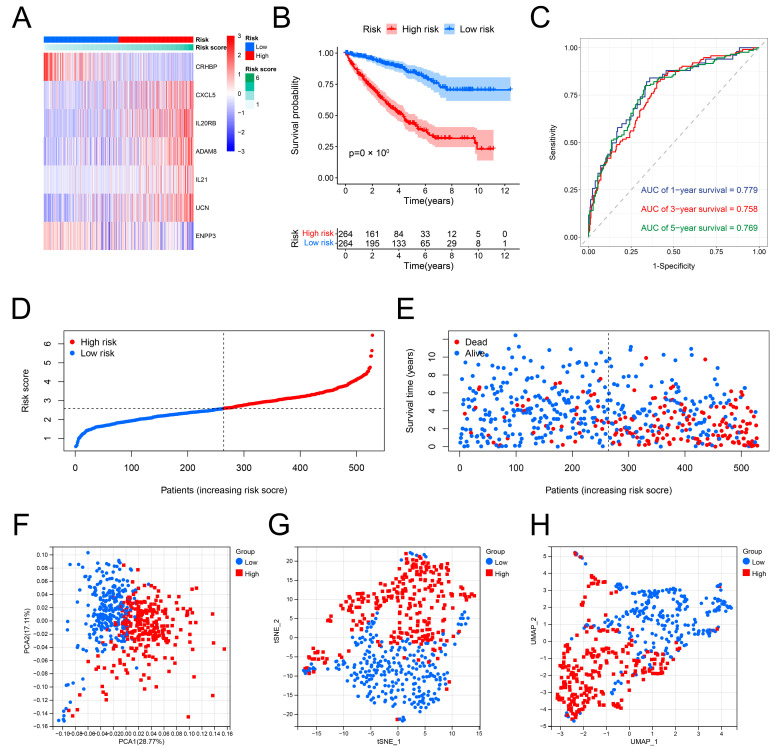
Predictive performance of the IRRS in the TCGA dataset. (**A**) Expression distribution of seven selected genes. (**B**) The difference in OS between the groups with high and low risk. (**C**) ROC curves of the IRRS for 1-, 3-, and 5-year OS prediction. (**D**,**E**) Distribution of ccRCC specimens with varying risk scores, survival times, and survival statuses. (**F**–**H**) Distribution of ccRCC specimens in the two risk groups was examined using PCA, t-SNE, and UMAP.

Internal validation was performed using the dataset from TCGA. The expression patterns of CRHBP, CXCL5, IL20RB, ADAM8, IL21, UCN, and ENPP3 in the high- and low-risk groups were shown on a heatmap. CXCL5, IL20RB, ADAM8, IL21, and UCN was up-regulated, whereas the expression of CRHBP and ENPP3 was down-regulated in the group with high risk (Figure 5A). When comparing the specimens from the group with low risk, it was found that the OS of the ccRCC specimens in the group with high risk was poorer (Figure 5B). Furthermore, the predictive accuracy of the IRRS for prognosis was assessed using the ROC curves. The respective AUCs for the 1-, 3-, and 5-year risk scores were 0.779, 0.758, and 0.769 (Figure 5C). According to the risk score plot, the prognosis of ccRCC specimens deteriorates with the increase in the risk scores (Figure 5D,E). Outcomes of the PCA, tSNE, and UMAP analyses showed the distribution of the two risk groups in distinct sections (Figure 5F–H). Subsequently, the external validation was carried out using the E-MTAB-1980 dataset. Expression patterns of seven genes linked to inflammation were shown on the heatmap (Figure S1A). The low-risk group exhibited a prognosis better than the other group (Figure S1B). The risk score’s respective AUCs for the 1-, 3-, and 5-year periods were 0.841, 0.771, and 0.765 (Figure S1C). The risk score plot confirmed that high-risk scores represented the poor OS of ccRCC specimens (Figure S1D,E). With the findings of the PCA, tSNE, and UMAP analyses, the ccRCC specimens could be classified into distinct sections (Figure S1F–H).

### 3.6. Relationship between the Risk Scores and Clinical Parameters

**Figure 6:**
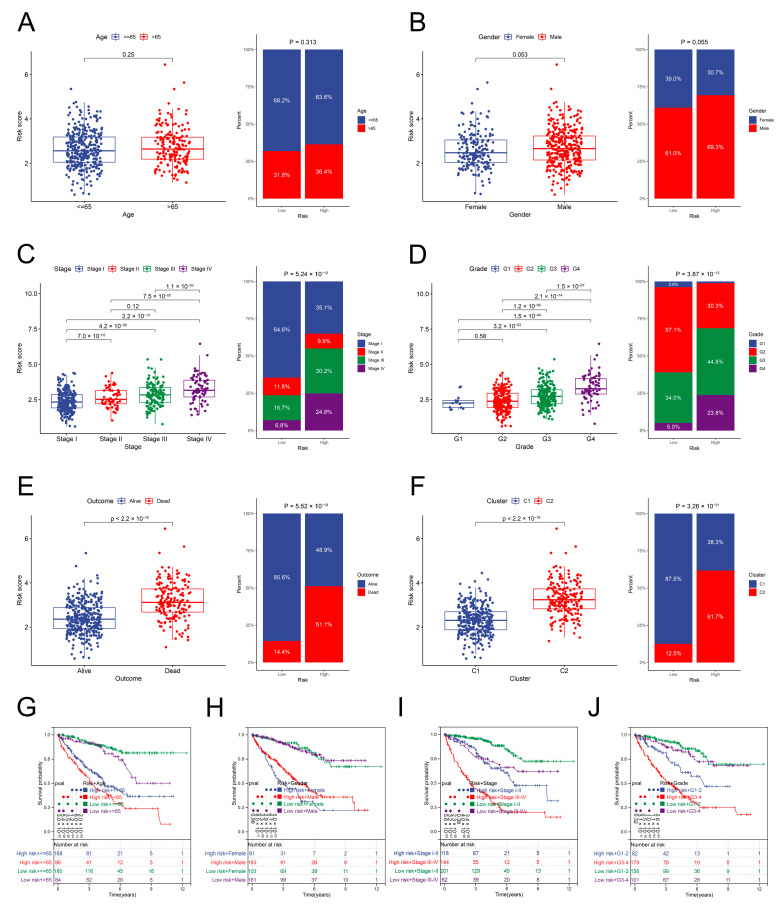
Clinical characteristics of the two risk groups in the TCGA dataset. (**A**–**F**) Distribution of the risk scores stratified by age, gender, TNM stage, tumor grade, survival status, and inflammation clusters, and the component proportion of clinical characteristics between the two groups. (**G**–**J**) OS of the ccRCC specimens of different risk groups and diverse clinical characteristics.

The risk scores of ccRCC specimens stratified by various clinical characteristics were compared. The composition of clinical parameters in both risk groups was explored as well. No variation was observed in the risk scores for the ccRCC specimens stratified by age and gender in the TCGA dataset (Figure 6A,B). The ccRCC specimens with advanced TNM stage and tumor grade and poor OS showed scores with high risk (Figure 6C–E). Notably, ccRCC specimens from the C2 cluster also showed scores with high risk. The low-risk group basically comprised the ccRCC specimens from the C1 cluster (Figure 6F). The K–M curves suggest that the risk score sustained a favorable prognostic value on the OS compared with the different clinical parameters (Figure 6G–J). Moreover, the external E-MTAB-1980 dataset was utilized to validate the relationship between the clinical characteristics and risk scores. The male ccRCC specimens exhibited higher risk scores than the female ccRCC specimens. Other results supported the outcomes of the TCGA dataset (Figure S2A–I).

### 3.7. Development and Validation of a Nomogram

**Figure 7:**
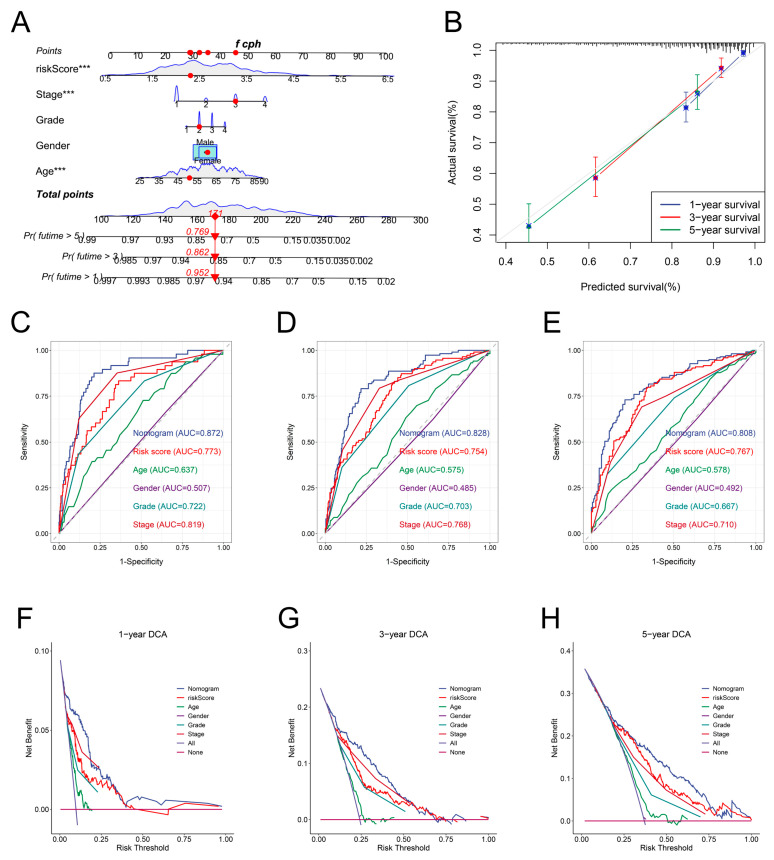
Development and internal validation of a nomogram. (**A**) Development of a nomogram based on the risk score, TNM stage, tumor grade, gender, and age. (**B**) Validation of the prediction effectiveness of the nomogram using calibration curves. (**C**–**E**) Comparing the ROC curves among the nomogram, risk score, age, gender, tumor grade, and TNM stage for 1-, 3-, and 5-year OS prediction. (**F**–**H**) Comparison of net benefits among the nomogram, risk score, age, gender, tumor grade, and TNM stage using DCA curves. *** *p* < 0.001.

We designed a nomogram by incorporating the IRRS and clinical parameters in the TCGA dataset to calculate the prognosis for ccRCC specimens accurately (Figure 7A). The nomogram’s calibration curves indicated that the predicted OS for the TCGA and E-MTAB-1980 datasets had a high uniformity with the actual value (Figure 7B and Figure S3A). The ROC curves of the nomogram showed better predictive accuracy than those of the IRRS and clinical parameters. In the TCGA dataset, the nomogram’s respective AUCs for 1-, 3-, and 5-year terms were 0.872, 0.828, and 0.808 (Figure 7C–E). For the E-MTAB-1980 dataset, the respective AUCs of the nomogram over 1, 3, and 5 years were 0.918, 0.910, and 0.879 (Figure S3B–D). According to the DCA curves, the nomogram offered optimal net benefits among all parameters (Figure 7F–H and Figure S3E–G).

### 3.8. The Immune Landscape of the IRRS Groups

**Figure 8:**
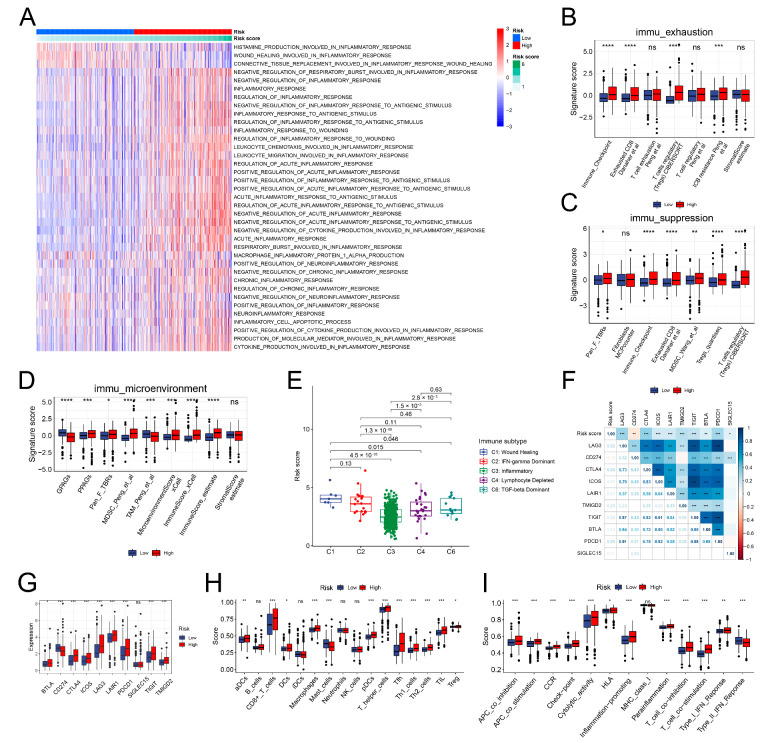
Immune characteristics between the two risk groups. (**A**) Functional enrichment of 37 inflammation-related BP terms from the different risk groups. (**B**) Immune exhaustion signature of both risk groups. (**C**) Immune suppression signature of both risk groups. (**D**) Immune microenvironment signature of both risk groups. (**E**) Risk score distribution in the different immune subtypes. (**F**,**G**) Correlation of the risk score with the inhibitory immune checkpoint genes. (**H**) Infiltration levels of the cells of the immune system in varying risk groups. (**I**) Correlation of the immune response processes with risk scores. * *p* < 0.05; ** *p* < 0.01; *** *p* < 0.001; and **** *p* < 0.0001.

The enrichment scores of 37 BP terms associated with inflammation in both risk groups were initially examined. Most of the processes associated with inflammation were activated in the high-risk group (Figure 8A). As the group with high risk was primarily composed of ccRCC specimens from the C2 cluster, it exhibited a similar immune-exhausted and -suppressed microenvironment as the C2 cluster (Figure 8B–D). Regarding the immune subtypes, most ccRCC specimens were of the inflammatory subtype. The inflammatory subtype had the lowest-risk scores, whereas the wound healing and the IFN-γ dominant subtypes exhibited the highest-risk scores (Figure 8E). Subsequently, we examined the link between the risk scores and inhibitory immune checkpoint molecules. The risk scores were positively linked to the inhibitory immune checkpoint molecules, except CD274 (Figure 8F). We did not observe variation in the SIGLEC15 expression levels between high- and low-risk groups. The expression of CD274 was down-regulated, and that of other inhibitory immune checkpoint molecules was up-regulated in the group with high risk (Figure 8G). Finally, the immune cell infiltration and response processes were analyzed using the ssGSEA algorithm. No differences were observed in the infiltration of B cells, immature dendritic cells (iDCs), neutrophils, and NK cells between the two risk groups. The infiltration of mast cells reduced in the group with high risk. The infiltration levels of the remaining types of immune cells were elevated in the high-risk group (Figure 8H). Multiple immune response processes, except the MHC class I and type II IFN responses, were enhanced in the high-risk group (Figure 8I).

### 3.9. Correlation between the IRRS and TMB

**Figure 9:**
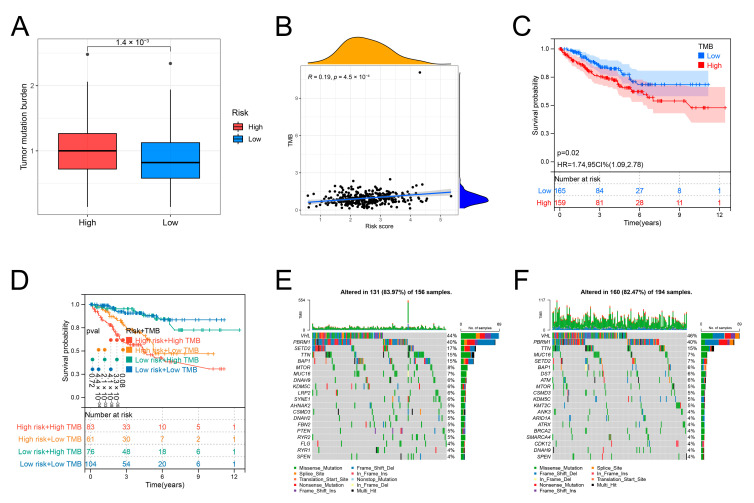
SNV signatures in the two risk groups. (**A**) TMB of both groups of risk. (**B**) Correlation of the TMB with the risk score. (**C**) OS of ccRCC specimens stratified using TMB. (**D**) OS of ccRCC specimens stratified by TMB and the risk score combination. (**E**) Tumor somatic mutation in the group with high risk. (**F**) Tumor somatic mutation in the group with low risk.

Compared to the group with low risk, the TMB was elevated in the other group (Figure 9A). The risk scores positively linked to the TMB (Figure 9B). According to the K–M curves, the ccRCC specimens with a high TMB have a poor prognosis (Figure 9C). Then, we combined the risk scores and TMB to predict OS of the ccRCC specimens. The risk scores and TMB showed evident prognostic values (Figure 9D). We further explored the SNVs between the two risk groups. VHL, PBRM1, and TTN were the genes that were mutated most frequently. SETD2 and BAP1 showed higher mutation frequencies in the group with high risk than the other group (Figure 9E,F, Table S1).

### 3.10. Correlation between the Low-Risk Group and Benefits of Targeted Therapy and Immunotherapy

**Figure 10:**
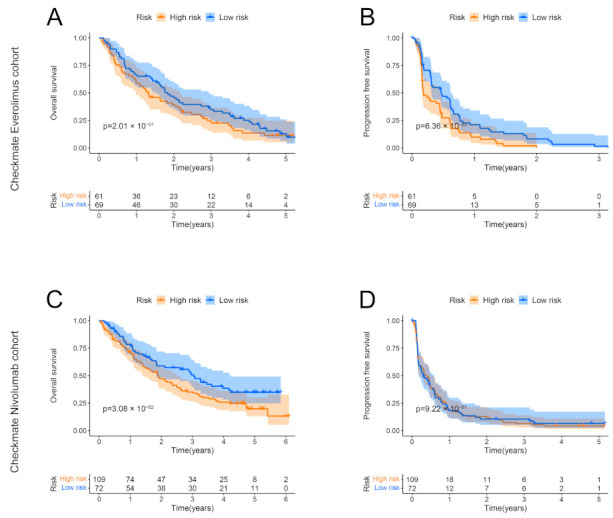
Validation of the IRRS in the Checkmate dataset. (**A**,**B**) OS and PFS of ccRCC patients treated with everolimus in both risk groups. (**C**,**D**) OS and PFS of ccRCC patients treated with nivolumab in both risk groups.

The Checkmate dataset was used to investigate the correlation between the IRRS and the prognostic benefits in patients with ccRCC treated with everolimus or nivolumab. In the everolimus cohort, low IRRS did not improve the OS but did improve the PFS (Figure 10A,B). In the nivolumab cohort, the OS of the group with high risk was remarkably worse than that of the group with low risk (Figure 10C); however, the PFS between the two groups did not differ (Figure 10D).

### 3.11. Identification of Potential Drug Molecules

**Figure 11:**
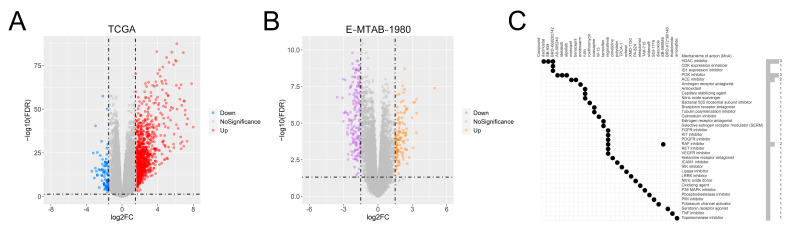
Prediction of potential drug molecules. (**A**) DEGs between both risk groups in the TCGA dataset. (**B**) DEGs between the two groups in the E-MTAB-1980 dataset. (**C**) Top 30 potential therapeutic targets and the corresponding MoAs.

For the identification of potential drug molecules, the DEGs between the two risk groups in both the TCGA and E-MTAB-1980 datasets were screened (Figure 11A,B). We considered the intersection of DEGs between the two datasets and identified 78 common up-regulated DEGs. These genes were used to perform CMap analysis. The top 30 potential drug molecules and their corresponding mechanisms of action (MoAs) were determined based on the connectivity scores (Figure 11C). Dacinostat, SB-939, and BRD-K68202742 served as histone deacetylase (HDAC) inhibitors; AS-605240, idelalisib, and alpelisib functioned as phosphatidylinositol 3-kinase (PI3K) inhibitors; moexipril and benazepril served as angiotensin-converting enzyme (ACE) inhibitors, and regorafenib and SB-590885 shared the MoAs of RAF inhibitors.

## 4. Discussion

Cancer initiation and progression are complicated stepwise processes driven by diverse factors. At present, limited strategies are available for the treatment of ccRCC and the evaluation of patient prognosis. Hence, there is a need to investigate the pathogenesis of ccRCC and find new therapeutic and prognostic biomarkers. Physiological inflammation is a protective process against harmful stimuli and is beneficial for maintaining tissue homeostasis [20]. However, the dysregulation of inflammation can induce DNA and protein damage, release reactive oxygen species, and activate oncogenes and oncogenic signaling pathways (9). These changes are involved in developing multiple diseases, including obesity, ischemia/reperfusion injury, and cancer. The findings of our study indicated that inflammatory signaling pathways, including the TNF-α, KRAS, and IL6 signaling pathways, were activated in the inflammation-active C2 cluster. In earlier studies, these inflammatory pathways have been linked to the onset and advancement of ccRCC. In addition, we found that inflammation enhanced hypoxia and CAF signatures, which emphasized its essential role in ccRCC.

TME is a complex ecosystem composed of various innate and adaptive immune cells together with cancer cells and the surrounding stroma [7]. Cancer and stromal cells attract and recruit immune cells to the site of infection by directly damaging tissues and releasing cytokines and chemokines. The recruited cells secrete inflammatory factors and amplify this response [21]. Hence, the balance in the proportion of immune cells is disrupted. Different immune cells can play tumor-promoting or tumor-suppressive roles and significantly influence clinical outcomes. Inflammation raised the infiltration levels of plasma cells, CD8 T cells, activated CD4 memory T cells, follicular helper T cells, Tregs, M0 macrophages, and neutrophils. Meanwhile, the infiltration levels of naive B cells, resting CD4 memory T cells, resting NK cells, monocytes, M1 macrophages, activated dendritic cells, resting mast cells, and eosinophils were reduced. Most notably, a high infiltration of CD8 T cells usually indicates an effective immune response against the tumor and a good prognosis in many cancers. However, CD8 T cells showed strong infiltration in the C2 cluster. This result might be partially supported by the fact that continuous antigen exposure induces the development of an exhausted phenotype. Inhibitory immune checkpoint molecules, including PD-1, CTLA4, and LAG3, are abundantly expressed on the surface of tumor-infiltrating lymphocytes, thus causing susceptibility to inhibitory signals from the TME [22]. In addition, infiltrating sufficient fully functional dendritic cells into tertiary lymphoid structures is necessary for CD8 T cells to be linked to a good prognosis in ccRCC [23]. Several studies have reported the dysfunction of dendritic cell maturation in ccRCC [24,25]. Consistent with this, our results indicated that the C2 cluster exhibited an immune-exhausted and immunosuppressive microenvironment and showed low infiltration of activated dendritic cells. Moreover, neutrophils are an important type of inflammatory cell, and their presence in ccRCC was associated with poor clinical outcomes [26]. Reportedly, the high infiltration of neutrophils impairs the prognosis of individuals with ccRCC who receive targeted therapy or immunotherapy [27,28]. However, the specific functions and mechanisms mediated by neutrophils in ccRCC remain unclear. According to investigations on other cancer types, neutrophils may exert a tumor-suppressive effect in early-stage tumors. In advanced-stage tumors, under the influence of an immunosuppressive TME, neutrophils are reprogrammed to a tumor-promoting phenotype characterized by an immature status, long life span, and low cytotoxicity [29,30]. Unlike the relatively less-studied neutrophils, the role of tumor-associated macrophages (TAMs) in the TME has been widely investigated. The generally accepted opinion is to classify TAMs into M1 and M2 subtypes. M1 macrophages exhibit an anti-tumor ability, whereas M2 macrophages contribute to cancer development. The subtype that TAMs polarize into depends on the stimulation of cytokines and chemokines. In ccRCC, an advanced tumor stage is accompanied by a high infiltration of M2 macrophages. Furthermore, the interaction between M2 macrophages and exhausted CD8 T cells is essential for establishing an immunosuppressive microenvironment [31]. These results indicate that inflammation may inhibit the tumor-suppressive ability of immune cells, thus promoting immune evasion in ccRCC.

An IRRS based on seven inflammation-related genes was constructed to comprehend the role of inflammation in ccRCC in a better way. The IRRS showed stable predictive performance for OS in ccRCC. Additionally, we identified the potential drug molecules and the corresponding MoAs according to the IRRS-classified high- and low-risk groups. The outcomes suggested that dacinostat, SB-939, and BRD-K68202742 shared the MoAs of HDAC inhibitors, and AS-605240, idelalisib, and alpelisib functioned as PI3K inhibitors. HDACs are a class of enzymes that catalyze the removal of acetyl groups from lysine residues on histone and non-histone proteins [32]. As HDACs are aberrantly up-regulated in various kidney diseases, they have attracted increasing attention in scientific research. Moreover, the relationship between HDAC inhibitors and inflammation has been reported. HDAC inhibitors block the NF-κB activation, thereby suppressing the transcription of various chemokines and cytokines that cause inflammation, including TNF-α, IL-1, and IL-6 [33]. In ccRCC, HDAC inhibitors are available for inhibiting ccRCC proliferation and enhancing the sensitivity of targeted therapies and immunotherapies [34,35,36]. However, the durable application of HDAC inhibitors can result in drug resistance owing to the overactivation of the serine/threonine protein kinase AKT [37]. PI3K, an upstream molecule of AKT, is essential for the activation of AKT. The combined inhibition of HDAC and PI3K synergistically induces the apoptosis of ccRCC cells [38]. Although the therapeutic efficacy of HDAC and PI3K inhibitors in ccRCC has been validated in the in vitro and in vivo experiments, further clinical trials are required to comprehensively estimate their therapeutic benefits.

As a preliminary investigation, some limitations should be noted. First, the IRRS was constructed based on a retrospective design. Although two independent datasets were utilized to verify the reliability of the IRRS, the IRRS requires further verification in prospective studies. Second, bioinformatics analysis was used to investigate the outcomes of this study. Comprehensive experiments would help better understand the characterization of inflammation in ccRCC.

## 5. Conclusions

This study comprehensively analyzed the differences among biological processes, the immune microenvironment, and the clinical outcomes between two inflammation subtypes. In addition, we constructed an IRRS based on seven inflammation-related genes. The IRRS showed good predictive performance for the clinical outcomes in patients with ccRCC. Lastly, we identified potential drug molecules for the treatment of ccRCC. The outcomes of our study might offer new perspectives on the identification of prognostic biomarkers and contribute to the improvement of treatment schemes.

## Data Availability

The datasets are available in the TCGA database (https://portal.gdc.cancer.gov/, accessed on 13 April 2022) as well as the ArrayExpress database (https://www.ebi.ac.uk/arrayexpress/, accessed on 27 December 2021).

## Supplementary Materials

The following supporting information can be downloaded at: https://www.mdpi.com/article/10.3390/genes13101897/s1, Figure S1. Predictive performance of the IRRS in the E-MTAB-1980 dataset. Figure S2. Clinical characteristics of the two risk groups in the E-MTAB-1980 dataset. Figure S3. Validation of the nomogram in the E-MTAB-1980 dataset. Table S1. The differences in the SNVs between high- and low-risk groups.
