# Supplementary figures and images for: Systematic Analysis of Immune Infiltration and Predicting Prognosis in Clear Cell Renal Cell Carcinoma Based on the Inflammation Signature
Source: Genes (Basel). 2022 Oct 19;13(10):1897. doi: 10.3390/genes13101897 (PMC9602121; doi:10.3390/genes13101897)

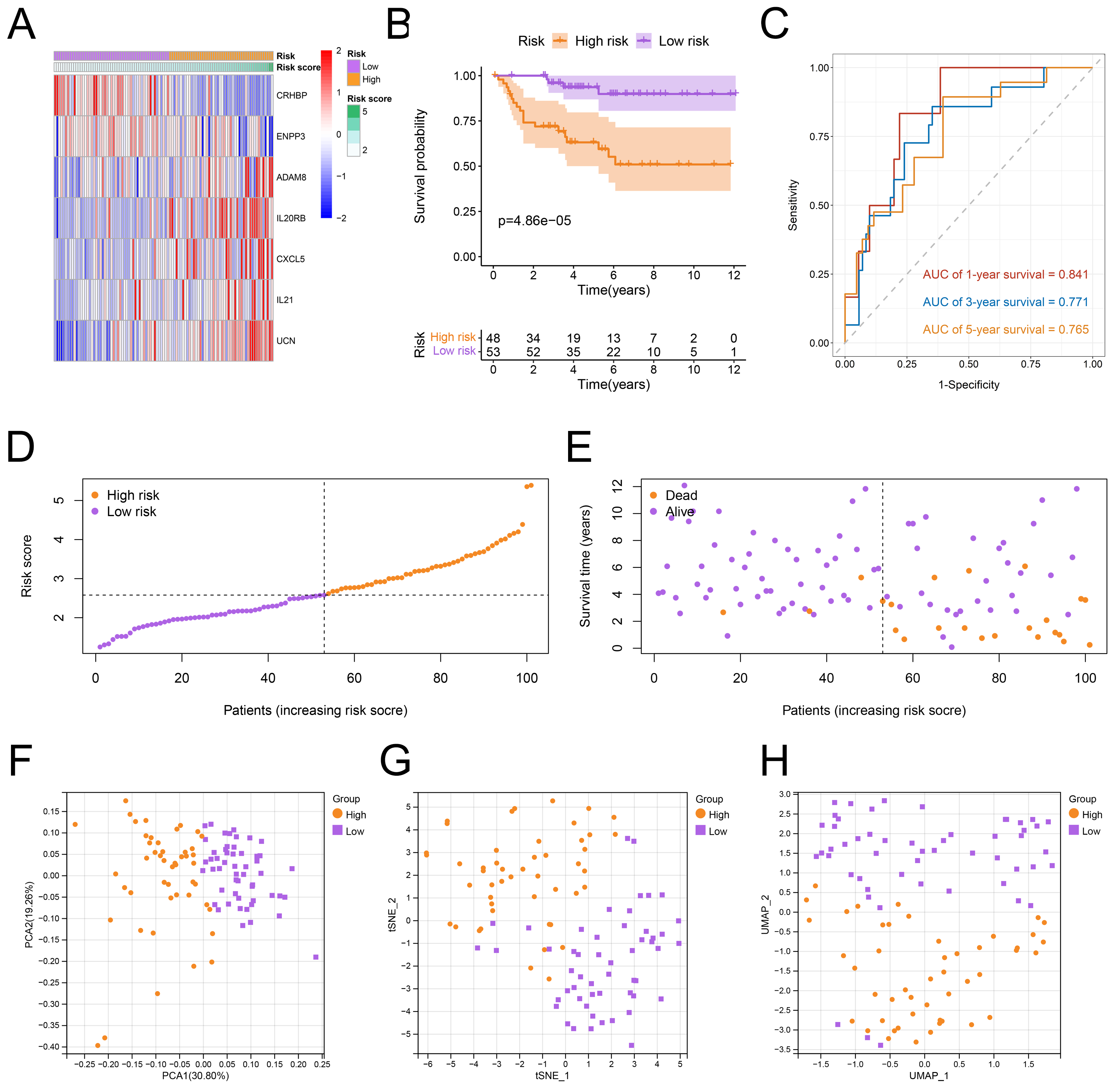

Supplement: Supplementary file 1 [file genes-13-01897-s001.zip › Figure S1.tif]

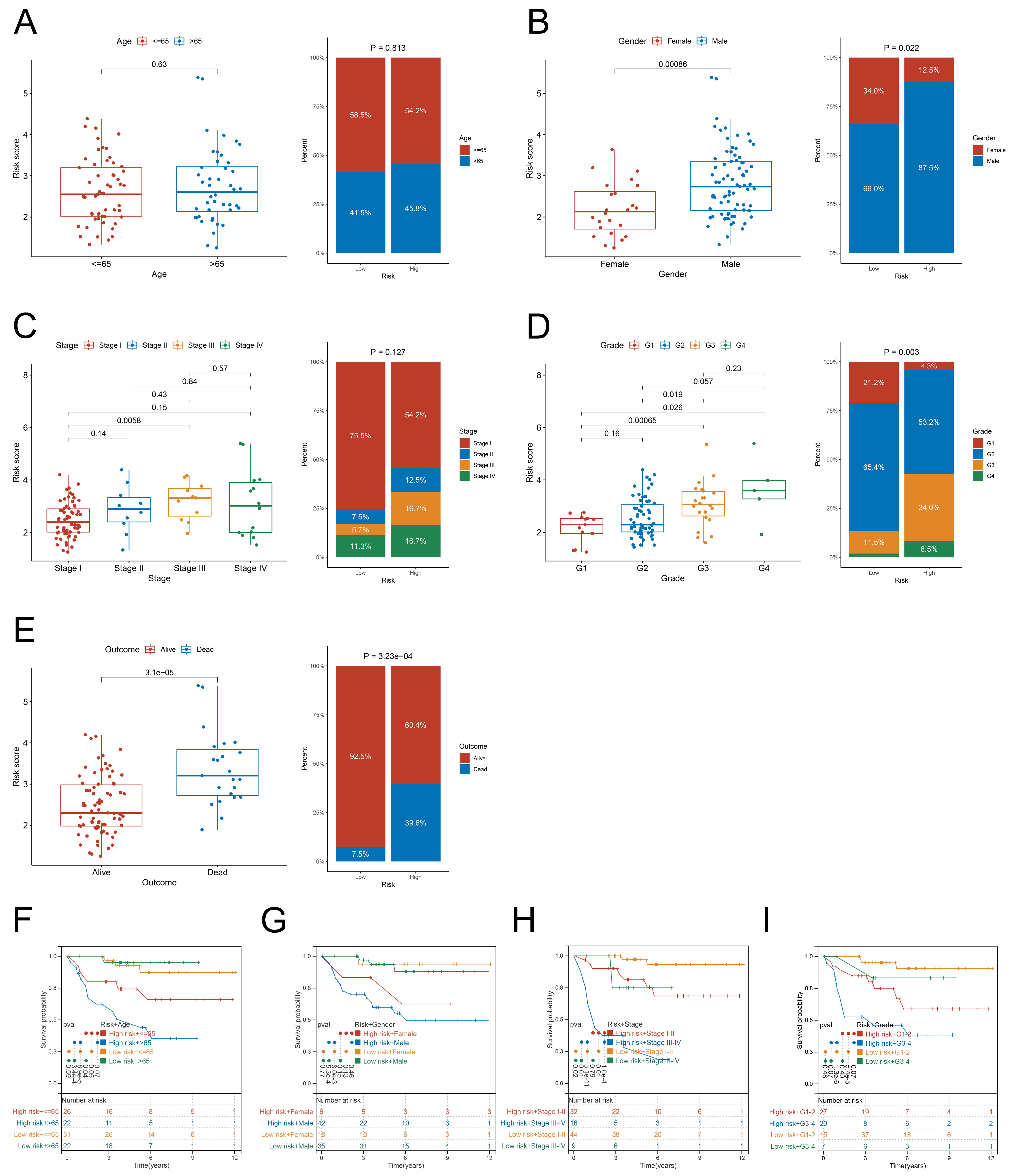

Supplement: Supplementary file 1 [file genes-13-01897-s001.zip › Figure S2.tif]

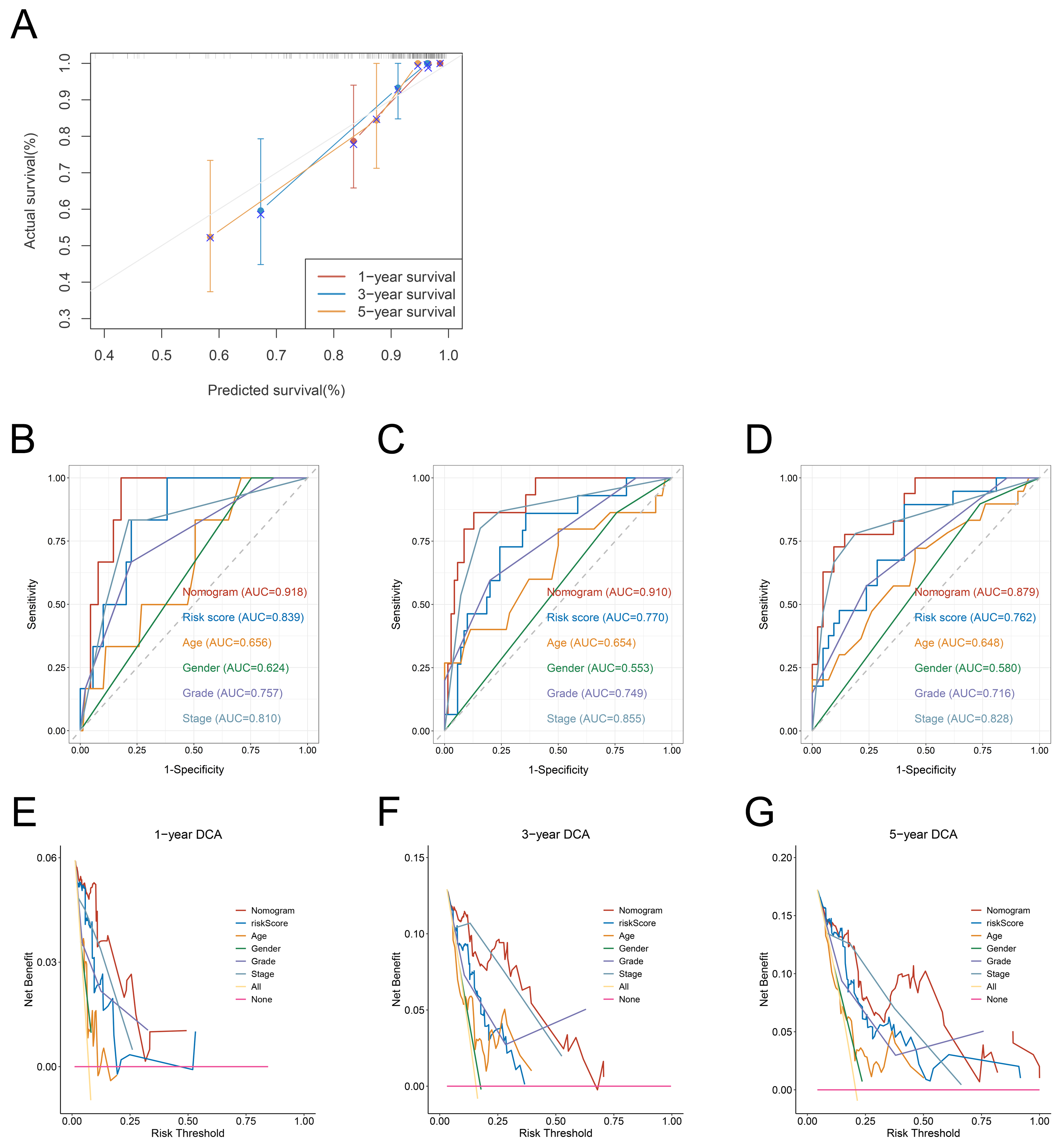

Supplement: Supplementary file 1 [file genes-13-01897-s001.zip › Figure S3.tif]
